# A quantitative analysis of spontaneous alternation behaviors on a Y-maze reveals adverse effects of acute social isolation on spatial working memory

**DOI:** 10.1038/s41598-023-41996-4

**Published:** 2023-09-07

**Authors:** Joowon Kim, Hyeyeon Kang, Young-Beom Lee, Boyoung Lee, Doyun Lee

**Affiliations:** 1 Center for Cognition and Sociality, Institute for Basic Science, Daejeon, 34126 Korea; 2 Graduate School of Medical Science and Engineering, Korea Advanced Institute of Science and Technology, Daejeon, 34141 Korea; 3 Department of Biomedical Engineering, College of Information and Biotechnology, Ulsan National Institute of Science and Technology (UNIST), Ulsan, 44919 Korea

**Keywords:** Neuroscience, Short-term memory, Spatial memory, Working memory, Animal behaviour

## Abstract

Animals tend to alternate between different choices, which requires the ability to remember recent choices. The Y-maze spontaneous alternation test is widely used in various animal models for assessing short-term memory, and its precise evaluation depends upon the accurate determination of the arm visit sequence. However, an objective method for defining arm visits is lacking owing to uncertainty regarding the extent to which an animal must go into the arm to be considered visited. Here, we conducted quantitative analyses on mice behavior in the Y-maze while systematically varying the arm visit threshold and assessed the effect of acute social isolation on spatial working memory. Our results revealed that 24-h social isolation significantly reduced spontaneous alternation rate when the arm threshold was set at the distal part of the arm. Furthermore, the memory of the recently visited arms faded away faster in the socially isolated mice. However, other behavioral factors were comparable to those of the group-housed mice, indicating a specific impairment of short-term memory. Our findings suggest that the location of arm visit threshold is critical for the precise evaluation of short-term memory, and our study provides a method for comprehensively and systematically assessing spontaneous alternation behavior in the Y-maze.

## Introduction

The spontaneous alternation test is widely used to study memory and motivation. This test relies on the natural tendency to alternate between cues or objects without explicit reward or punishment^1–5^. It is extensively used in assessing spatial working memory, which involves the ability to hold information about the spatial environment over a short period, enabling individuals to remember and navigate through their physical surroundings^6–12^. Among the multiple ways to perform the spontaneous alternation test, the Y-maze test is commonly used because it does not require handling or training and is less likely to induce stress and fear^5,13^. It has become an essential tool for the behavioral screening of various animal models.

The Y-maze comprises three arms positioned 120 degrees apart, which allows rodents to explore freely. Rodents naturally tend to explore the arm that was not visited immediately before, resulting in sequential visits to all three arms. A high percentage of alternation has been considered to be indicative of good spatial memory^5,8,9,11,12,14^. Traditionally, the analysis of spontaneous alternation behavior on the Y-maze has relied on visual scoring, which entails calculating the rate of choosing the less recently visited arm and counting the number of arm entries to compare locomotive activity between the control and experimental groups^5,8,9,11,12,14^. However, manually performing these processes is labor-intensive and prone to errors and biases. To address this issue, attempts have been made to use infrared sensors installed at the arm to monitor the sequence of arm entries^7,13,15–17^. While these approaches provide objective and reproducible assessments of spontaneous alternation rates, they do not directly measure other behaviors such as locomotive behaviors.

Recent advances in video tracking technology have enabled the analysis of various behavioral aspects besides arm entry and alternation rates. These technologies offer contour-based tracking with millisecond resolution, enhancing the accuracy of locomotive behavior analysis^18,19^. Furthermore, applying deep learning techniques has greatly improved the accuracy of body point detection, enabling the tracking of multiple body points and the analysis of a rich behavioral repertoire^20–23^. Although high spatiotemporal resolution tracking data are readily available, most analyses of behaviors on Y-mazes have been restricted to alternation rates and the number of arm entries. Only a few studies have extended this analysis to include the time spent in each arm and the center zone^12,13^.

Accurately estimating the spontaneous alternation rate on the Y-maze relies heavily on determining the precise arm visit sequence. However, when rodents enter an arm, they frequently do not explore it to the distal end, often turning around in the middle. It is unclear whether rodents consider the partially explored arm to be visited and, therefore, are less likely to choose it again. Currently, there is no definitive method for evaluating spontaneous alternation behavior, with different studies using various criteria to determine whether an animal has visited an arm. Some studies define a visit as when the animal places all four paws within the perimeters of an arm^24–28^, while others define a visit as reaching the middle or distal third of an arm^13,29^. These methods are ambiguous and depend too heavily on observer objectivity. Moreover, the use of different arm thresholds has not been adequately justified, and how different thresholds affect the calculation of the alternation rate has not been systematically examined.

Moreover, high spatiotemporal resolution tracking data facilitate the analysis of various behavioral factors on the Y-maze, such as travel distance, time spent in each arm, and the time delay between consecutive arm visits. However, the influence of these behavioral factors on the spontaneous alternation rate remains unexplored. This is particularly important because experimental manipulation and treatment may alter one of these behavioral factors, resulting in a change in the spontaneous alternation rate without directly affecting short-term memory. Therefore, this study aims to examine the effect of varying the arm visit threshold on the spontaneous alternation rate calculation and to investigate the impact of different behavioral factors on the spontaneous alternation rate.

We used our newly developed analysis pipeline to assess the effect of acute social isolation on short-term memory. The recent rise in reduced social interaction, such as social distancing due to the Covid-19 pandemic, has highlighted the adverse effects of social isolation on the mental health of the general population. Several studies have reported various effects of social distancing, such as anxiety and depression^30,31^, as well as negative effects on the mental health of children^32,33^. While acute social isolation has been shown to impair social memory in rodents^34–38^, its effects on spatial working memory have not been thoroughly investigated. To address this gap, we used the Y-maze spontaneous alternation test and our newly developed analysis pipeline to assess the adverse effects of acute social isolation on spatial working memory.

## Results

### Defining center zone boundary based on the occupancy of body positions

**Figure 1:**
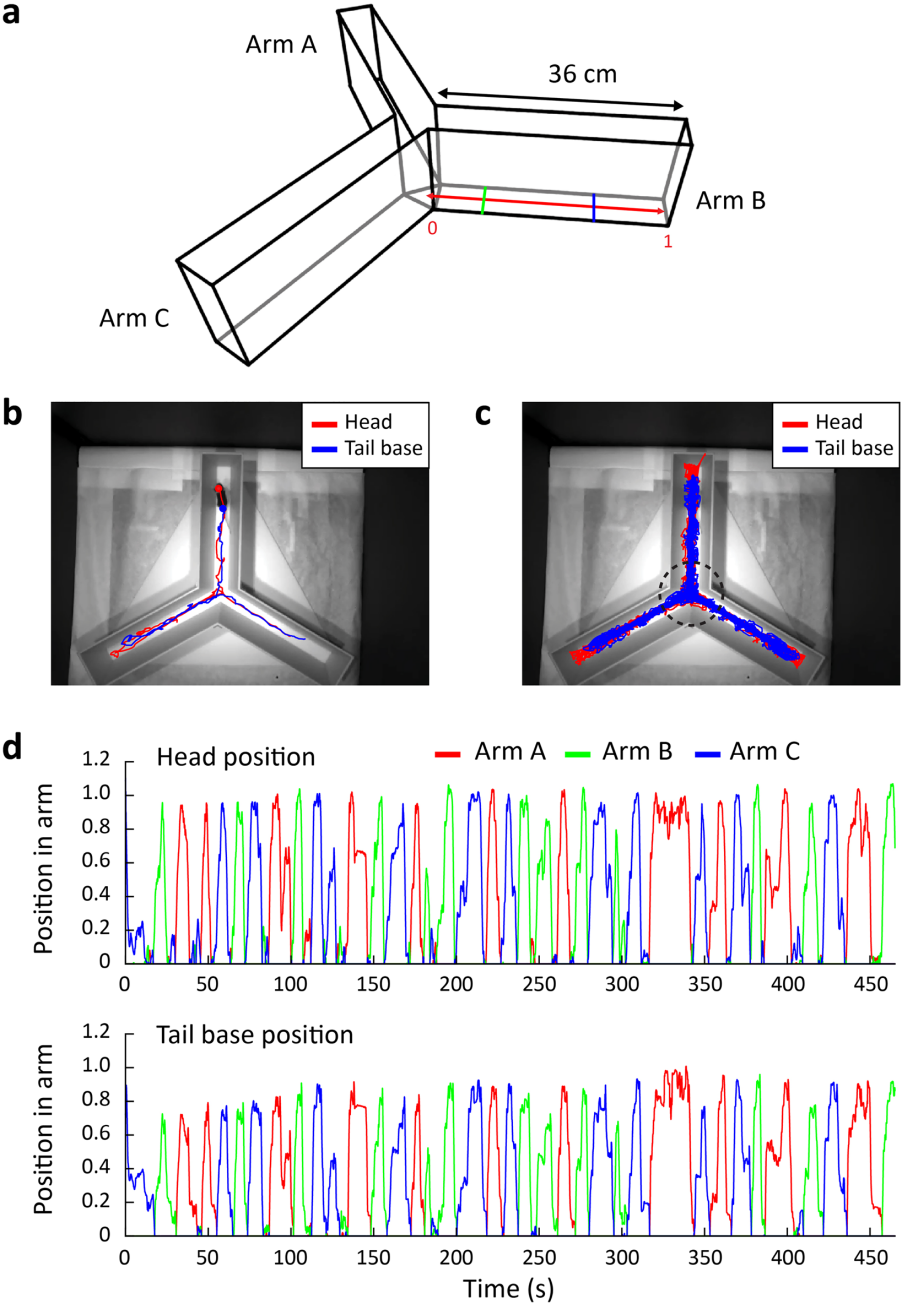
Tracking the trajectory of the head and tail base of mice in the Y-maze. (**a**) The structure of the Y-maze. The red line indicates the normalized position of the arm. The position on the arm was normalized to the length of the arm, so that the location of 0 was the center of the Y-maze and the location of 1 was the end of the arm. The blue and green lines indicate locations of 0.7 and 0.25, respectively. (**b**) A segment of the head and the tail base trajectories. (**c**) Trajectory of a mouse throughout a session. The perimeter of the circle corresponds to an arm location of 0.25. (**d**) The position of the head (top) and tail base (bottom) relative to the normalized coordinates of each arm. Different arms are indicated by different colors.

To quantitatively analyze the spontaneous alternation behavior of mice on a Y-maze (Fig. 1a), we utilized the automatic animal tracking software, DeepLabCut^20^, to track the position of their head and the tail base at 30 Hz (Fig. 1b). Using the tracking data, we reconstructed the entire trajectory of the mice in the Y-maze (Fig. 1c). Subsequently, the positions of the head and tail base were projected onto the axis of the arm. The normalized position trajectory along the arm axis showed that the example mouse thoroughly explored all three arms of the Y-maze (Fig. 1d).

**Figure 2:**
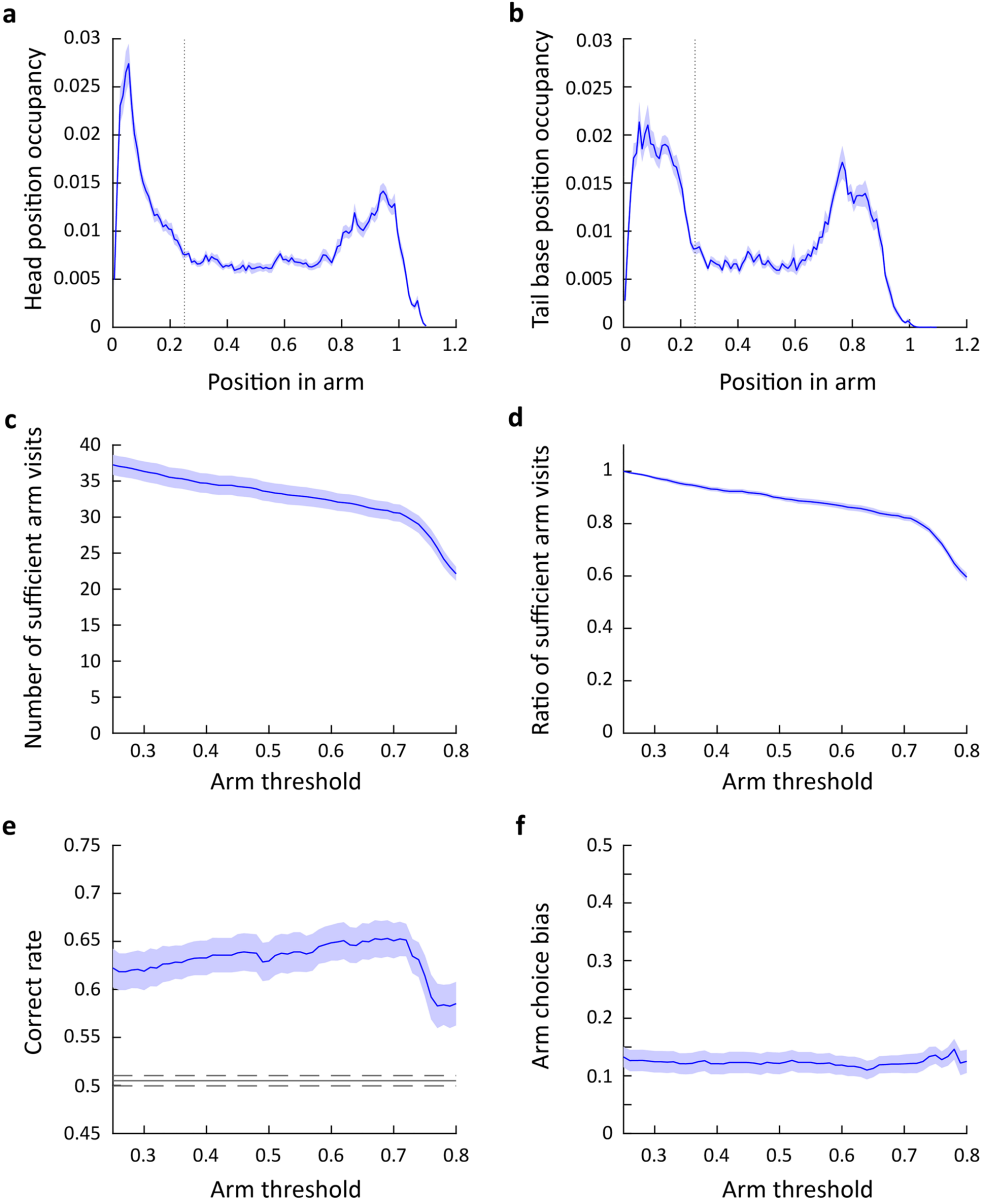
Determination of center zone boundary and assessment of arm visit and alternation rate with varying arm visit thresholds. (**a**) Occupancy of the head position at each position in the arm (mean ± SEM (shade), n = 24 mice). (**b**) Occupancy of the tail base position at each position in the arm (mean ± SEM (shade), n = 24 mice). (**c**) The change in the number of sufficient arm visits by the change in the arm threshold (mean ± SEM (shade), n = 24 mice). (**d**) The change in the ratio of sufficient arm visits by the change in the arm threshold (mean ± SEM (shade), n = 24 mice). (**e**) The change in the correct rate by the change in the arm threshold (mean ± SEM (shade), n = 24 mice). The gray line indicates the chance level spontaneous alternation rate calculated using the arm choice bias in (f). The dashed lines represent 95% confidence interval. (**f**) The change in arm choice bias by the change in the arm threshold (mean ± SEM (shade), n = 24 mice).

Y-mazes typically comprise three identical rectangular arms conjoined at the center, forming a small triangle, and do not usually have a clearly defined center area. Previous studies have considered the threshold for arm entries to be the border between the triangle and the arm. However, we observed that the mice exhibited extensive explorative behaviors not only around the triangular center area, but also in the initial portion of the arms. Therefore, using the triangular center area as a reference for defining arm entry and exit was inadequate. Instead, we established an objective boundary for the center of the maze using increased occupancy in the center area. Occupancy levels of the head and the tail base were plotted across 24 mice, revealing a high degree of occupancy at the center of the Y-maze. As the mice moved away from the center, the occupancy of both the head and tail base gradually decreased, reaching a steady level at approximately 25% of the arm length from the center. Therefore, we defined this location as the boundary of the center zone (Fig. 1a and 2a,b). We defined arm entry and exit as the moment when both the head and the tail base crossed the boundary of the center zone.

### Assessment of arm visits and spontaneous alternation rate with varying arm visit thresholds

Our observations showed that mice tend to fully explore an arm once they enter it. However, there were also cases where they exited an arm before reaching the end of the arm (Fig. 1d), making the objective definition of an arm visit unclear. To determine whether a visit to an arm is sufficient to be considered visited, we need to set criteria for how far into an arm the mice need to travel. We established a threshold between the center zone and the distal end of an arm, referred to as the arm visit threshold. An arm visit was considered sufficient if both the head and tail base of the mice crossed this threshold.

We increased the threshold by increments of 0.01 and observed a gradual decrease in the number and ratio of sufficient arm visits until the arm threshold reached approximately 0.7 (Fig. 2c,d). In addition, the correct rate, which measures the frequency at which the mice selected the arm they did not travel in the last and second last arm visits, gradually increased (Fig. 2e). Since rodents exhibit a turning bias when choosing between the left and right arms, spontaneous alternation may arise as a result of the bias rather than a memory of the previous arm visits. Thus, we evaluated the arm choice bias, i.e., the absolute difference between the ratio of selecting the left and right arms and 0.5, and its contribution to the spontaneous alternation behavior. We found that the mice showed a preference for selecting the arm on their preferential side, and the arm choice bias did not significantly differ across the range of the arm threshold (Fig. 2f). Next, we simulated spontaneous alternation rates by assuming that mice navigated the arms purely based on the arm choice bias. The simulated alternation rate was substantially below the observed spontaneous alternation rate (Fig. 2e), suggesting the turning bias alone cannot explain the spontaneous alternation behavior on the Y-maze.

**Figure 3:**
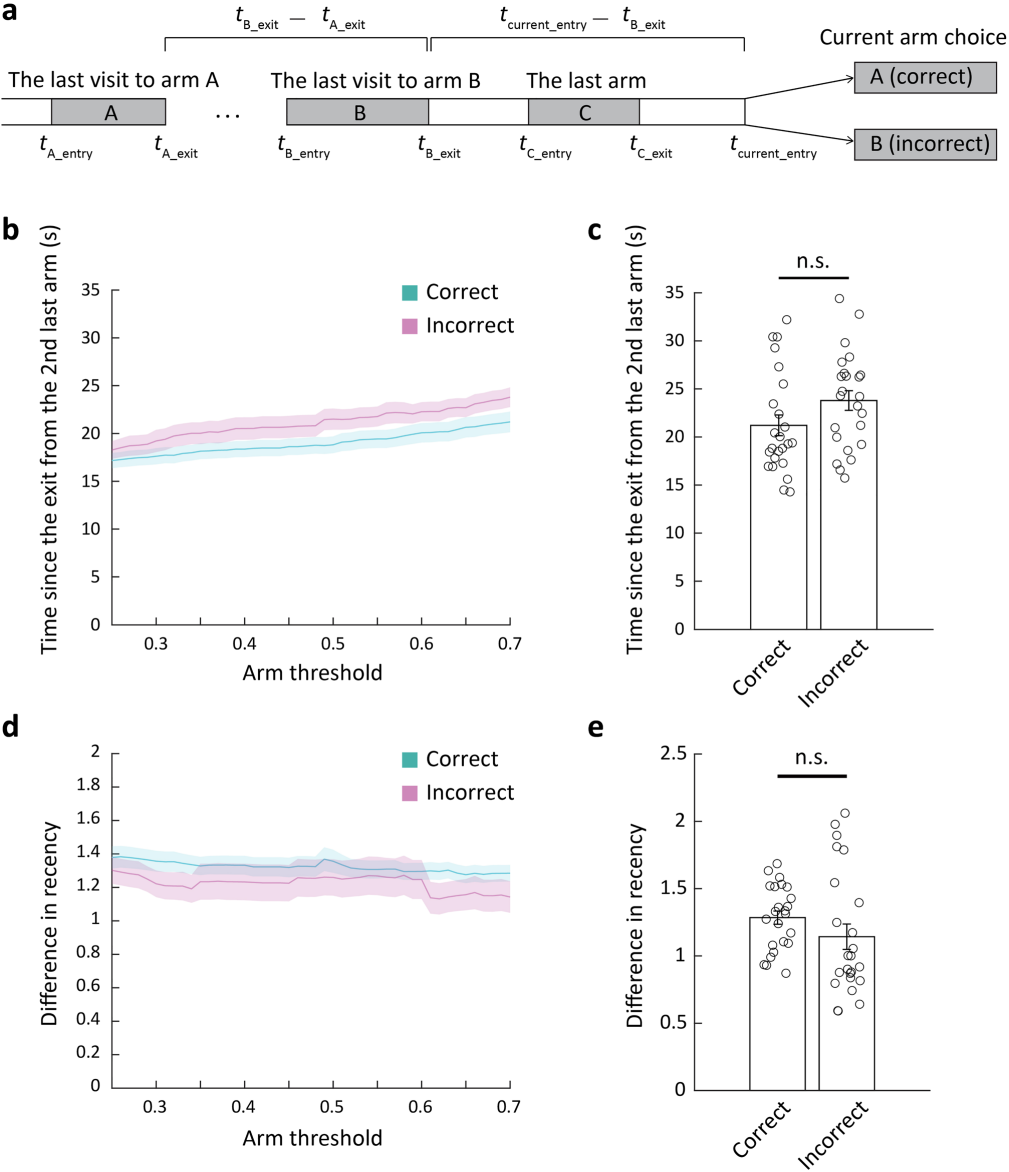
Maintenance of short-term memory for previously visited arms in group-housed mice during spontaneous alternation. (**a**) Schematic drawing of the time parameters. (**b**) The change in the time since the exit from the 2^nd^ last-visited arm by the change in the arm threshold for correct and incorrect trials (mean ± SEM (shades), n = 24 mice). (**c**) Comparison of the time since the exit from the 2^nd^ last-visited arm between correct and incorrect trials at arm threshold of 0.7 (two-sided unpaired t-test, n = 24, *p* = 0.089). (**d**) The change in the difference in recency by the change in the arm threshold for correct and incorrect trials (mean ± SEM (shades), n = 24 mice). (**e**) Comparison of the difference in recency between correct and incorrect trials at arm threshold of 0.7 (two-sided unpaired t-test, n = 24, *p* = 0.66).

Our results indicate that the correct rate varies depending on which criteria are used. However, it decreased abruptly when the arm threshold was set beyond 0.7. This is mainly because when both the head and tail base positions were above 0.7, the mice reached near the end of the arm. This speculation is supported by the following observations: (1) the occupancy of the head and tail base peaked around the arm position of 0.7–1.0, indicating that the mice have reached the end of an arm and was exploring rather than traveling (Fig. 2a,b), (2) the number of sufficient arm visits displayed a steep decrease after the arm threshold of 0.7 (Fig. 2c,d), and (3) the correct rate peaked at around the arm threshold of 0.7 and rapidly decreased thereafter (Fig. 2e). Therefore, we varied the arm threshold between 0.25 and 0.7 as the appropriate threshold values to be tested in the subsequent analyses.

### Retention of short-term memory for previously visited arms in group-housed mice during spontaneous alternation

The Y-maze spontaneous alternation test is a common method for assessing short-term memory, which refers to the ability to retain information for a brief period. In this test, if mice spend a prolonged time on an arm, they are more likely to forget which arm they visited previously. Therefore, we examined the effect of the time elapsed since the mice exited from the previously visited arms on the current arm choice (Fig. 3a). We first examined whether the time elapsed since the exit from the second last-visited arm (*t*_current_entry_—*t*_B_exit_) affected spontaneous alternation. The time elapsed since the exit from the second last-visited arm did not differ depending on whether the current arm choice was correct or incorrect (Fig. 3b,c). When the mice exited an arm and were about to choose between the other two arms, they tended to select the arm they had visited a longer time ago. Consequently, the difference in recency between the two arms becomes a critical factor, which can be evaluated by the difference between the times elapsed since the last exit from those two arms (*t*_B_exit_—*t*_A_exit_) normalized by the time since the last exit from the current incorrect arm (*t*_current_entry_—*t*_B_exit_). We excluded the rare cases in which the mice reentered the same arm they had just exited from the analysis. Thus, the current incorrect choice was always the second last-visited arm. The analysis revealed no difference in the difference in recency between the correct and incorrect choices (Fig. 3d,e). These results indicate that the time elapsed since the mice exited from the second last-visited arm or the difference in recency between the correct and incorrect arms did not significantly affect their current arm choice, suggesting that the memory of the previously visited arms did not fade away during this time period.

**Figure 4:**
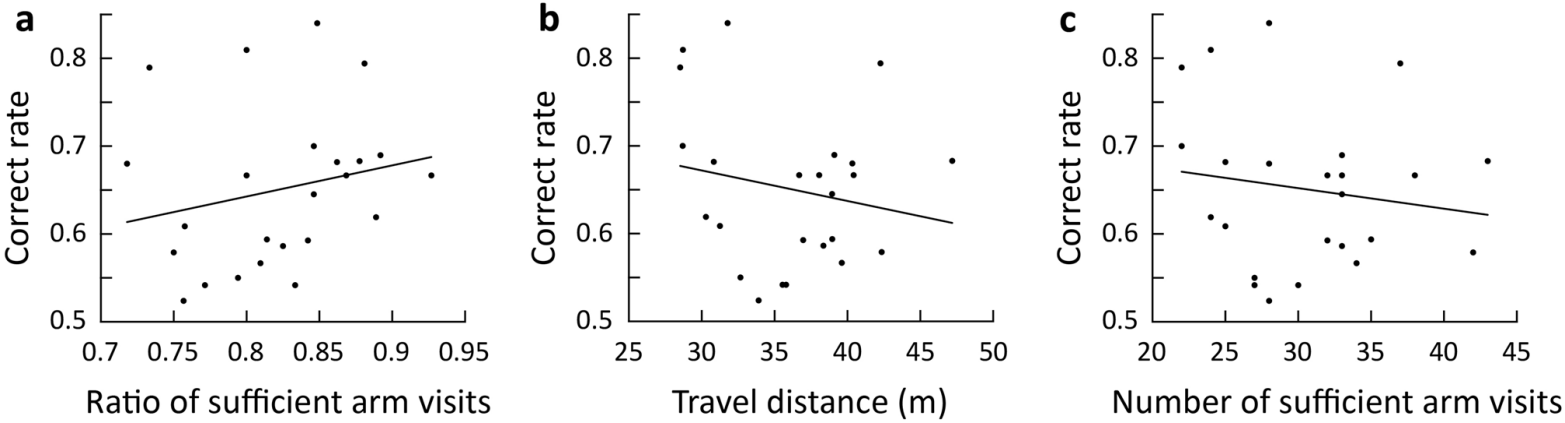
Effects of various locomotive parameters on the correct rate. (**a**) Correlation between the ratio of sufficient arm visits and the correct rate (n = 24 mice, Pearson’s *r* = 0.22, *p* = 0.31). (**b**) Correlation between the travel distance and the correct rate (n = 24, Pearson’s *r* =  − 0.19, *p* = 0.36). (**c**) Correlation between the number of sufficient arm visits with an arm threshold of 0.7 and the correct rate (n = 24, Pearson’s *r* =  − 0.15, *p* = 0.48).

In addition, we investigated the effects of various other factors on the correct rate. We found no significant correlation between the ratio of sufficient arm visits (crossing the arm threshold) to arm entries (crossing the boundary of the center zone) and the correct rate (Fig. 4a). In addition, the travel distance of the mice during the session did not demonstrate a significant correlation with the correct rate (Fig. 4b), nor did the number of sufficient arm visits (Fig. 4c). These results indicate that various factors, including the number and ratio of sufficient arm visits and the travel distance, are not correlated with the correct rate.

### Detrimental impact of acute social isolation on spatial working memory

**Figure 5:**
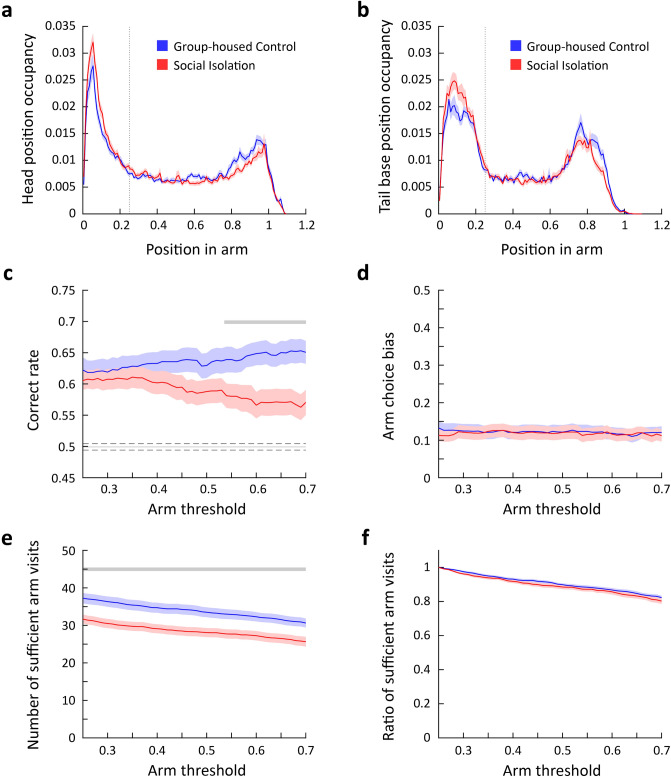
Negative impact of 24-h social isolation on spontaneous alternation behavior. (**a**) Comparison of the head position occupancy across the position in the arm between the GC and SI cohorts (mean ± SEM (shades), n = 24 for GC, n = 28 for SI). (**b**) Comparison of the tail base position occupancy across the position in the arm between the GC and SI cohorts (mean ± SEM (shades), n = 24 for GC, n = 28 for SI). (**c**) Comparison of the correct rate across the arm threshold between the GC and SI cohorts (mean ± SEM (shades), n = 24 for GC, n = 28 for SI). The thick gray line indicates significant sections (cluster-based permutation test, two-sided, *p* < 0.05). The thin gray line indicates the chance level spontaneous alternation rate calculated using the arm choice bias in (d). The dashed lines represent 95% confidence interval. (**d**) Comparison of the arm choice bias between the GC and SI cohorts (mean ± SEM (shades), n = 24 for GC, n = 28 for SI). (**e**) Comparison of the change in the number of sufficient arm visits by the arm threshold between the GC and SI cohorts (mean ± SEM (shades), n = 24 for GC, n = 28 for SI). The gray line indicates significant sections (cluster-based permutation test, two-sided, *p* < 0.05). (**f**) Comparison of the change in the number of sufficient arm visits by the arm threshold between the GC and SI cohorts (mean ± SEM (shades), n = 24 for GC, n = 28 for SI).

To assess the effect of acute social isolation on spatial working memory, a group of mice was single-housed for 24 h (i.e., socially isolated acutely) before the Y-maze spontaneous alternation test (social isolation [SI] cohort). We compared their performance to that of a control group (group-housed control [GC] cohort). The SI cohort showed similar head and tail base position occupancies, justifying the use of the same center zone threshold of 0.25 (Fig. 5a,b). When we varied the arm visit threshold from 0.25 to 0.7, the correct rate of the SI cohort gradually decreased along the arm threshold in contrast to the GC cohort, which showed a gradual increase in the correct rate along the increasing arm threshold. As a result, the SI cohort showed a significantly lower correct rate than that of the GC cohort when the arm threshold exceeded 0.54, indicating that acute social isolation negatively affects short-term memory (Fig. 5c). Arm choice bias showed no significant difference between the GC and SI cohorts for the entire arm threshold range, indicating that the reduction in the correct rate in the SI cohort cannot be explained by an altered arm choice bias (Fig. 5d). In addition, the number of sufficient arm visits showed a significant difference between the GC and SI cohorts across the arm threshold range (Fig. 5e), while the ratio of sufficient arm visits showed no difference between the two cohorts (Fig. 5f). These results suggest that in order to accurately demonstrate the effect of experimental manipulations on short-term spatial memory, it is necessary to analyze spontaneous alternation behaviors while varying the arm visit thresholds.

**Figure 6:**
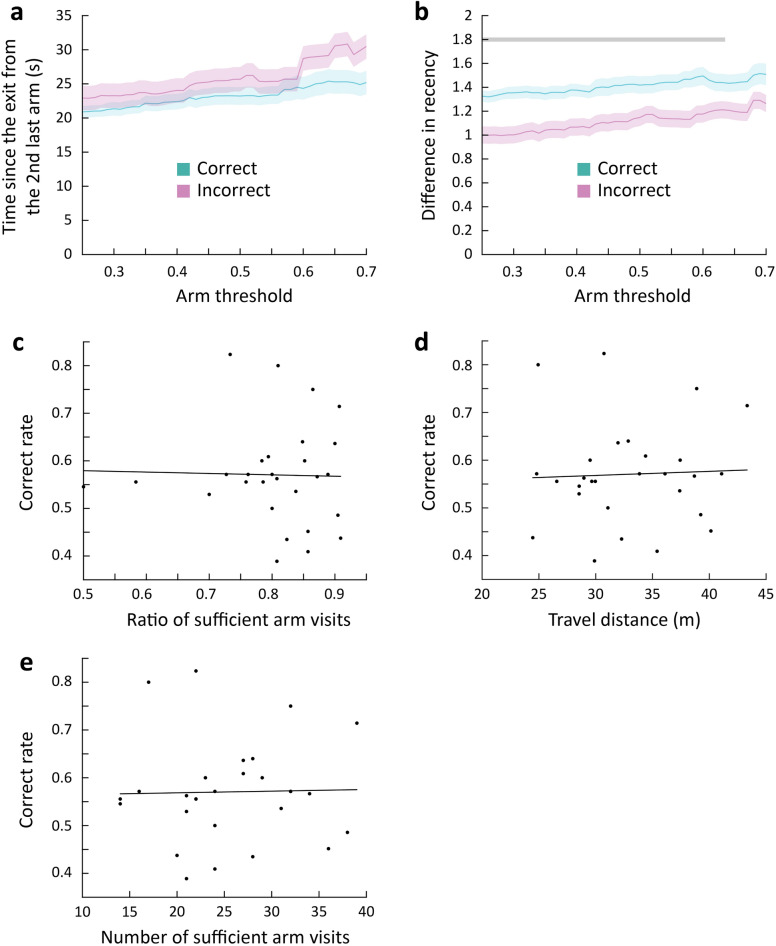
Impaired memory of recently visited arms and lack of correlation between various locomotive parameters and the correct rate in the SI cohort. (**a**) The change in the time since the exit from the 2^nd^ last arm by the change in the arm threshold for correct and incorrect trials (mean ± SEM (shades), n = 28). (**b**) The change in the difference in recency by the change in the arm threshold for correct and incorrect trials (mean ± SEM (shades), n = 28). The gray line indicates significant sections (cluster-based permutation test, two-sided, *p* < 0.05). (**c**) Correlation between the ratio of sufficient arm visits and the correct rate (n = 28, r =  − 0.025, *p* = 0.90). (**d**) Correlation between the travel distance and the correct rate (n = 28, r = 0.042, *p* = 0.83). (**e**) Correlation between the number of sufficient arm visits with an arm threshold of 0.7 and the correct rate (n = 28, r = 0.022, *p* = 0.91).

In addition, we investigated whether the memories of the recently visited arms decay in a quicker rate in the SI cohort compared to the CG cohort. The time elapsed since the exit from the second last-visited arm did not differ between correct and incorrect arm choices in the SI cohort (Fig. 6a). However, we observed that the difference in recency was significantly lower for the incorrect choices in the SI cohort (Fig. 6b). This result suggests that the memory of the recently visited arms faded away faster in the SI cohort than the CG cohort.

Similar to the GC cohort, we considered various parameters, and their effects on the correct rate in the SI cohort. The ratio of sufficient arm visits, travel distance, and the number of sufficient arm visits did not significantly correlate with the correct rate in the SI cohort (Fig. 6c–e). These results indicate that the various parameters that did not affect the correct rate in the GC cohort also had no effect on the correct rate in the SI cohort.

**Figure 7:**
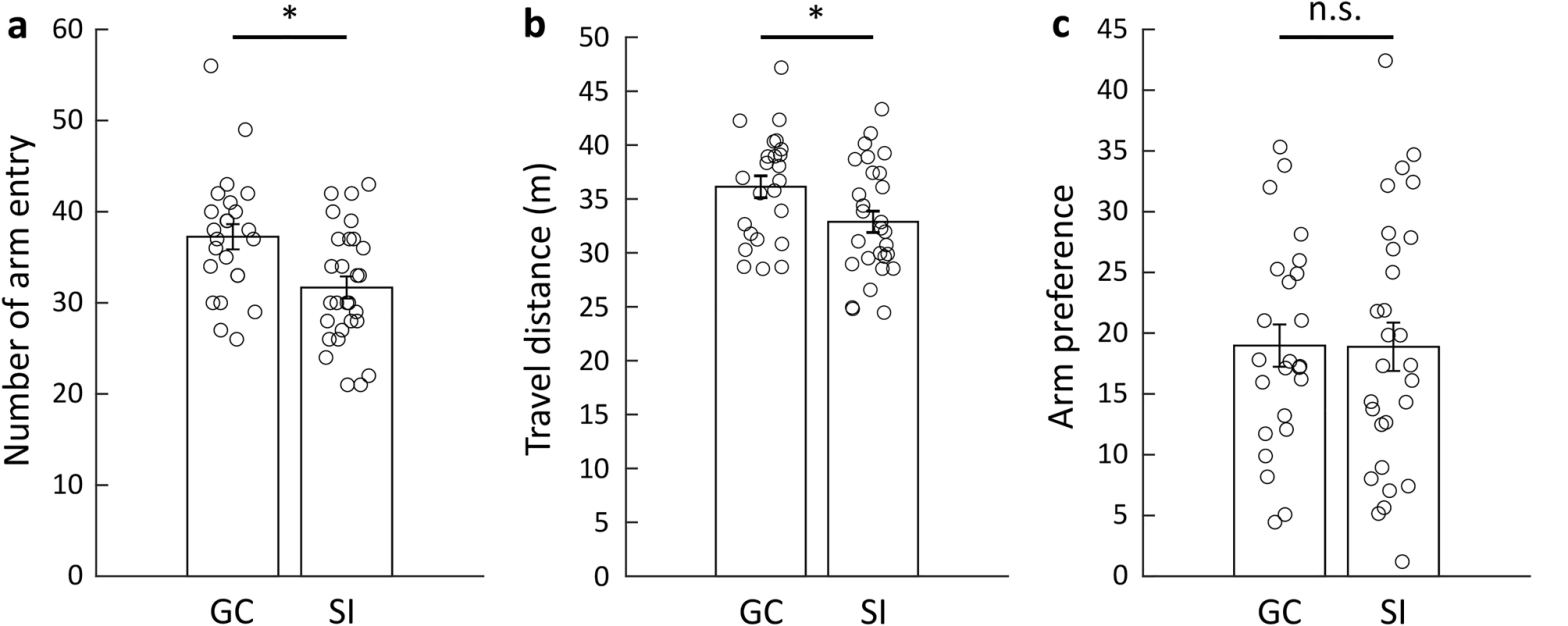
Differences in locomotive behaviors and arm preference in the Y-maze between the GC and SI cohorts. (**a**) Comparison of the number of arm entries between the GC and SI cohorts (unpaired t-test, n = 24 for GC, n = 28 for SI, *p* = 0.0038). (**b**) Comparison of the travel distance between the GC and SI cohorts (unpaired t-test, n = 24 for GC, n = 28 for SI, *p* = 0.027). (**c**) Comparison of the arm preference between the GC and SI cohorts (unpaired t-test, n = 24 for GC, n = 28 for SI, *p* = 0.97).

We also compared other behavioral factors between the GC and SI cohorts and found that the SI cohort had decreased arm entries and travel distance compared to the GC cohort (Fig. 7a,b). In addition, there was no significant difference in arm preference (i.e., the standard deviation of the duration spent in each arm) between the GC and SI cohorts (Fig. 7c). Overall, these results suggest that 24-h social isolation diminishes spontaneous alternation behavior due to short-term memory impairment rather than changes in other behaviors.

## Discussion

A typical Y-maze comprises three rectangular arms connected at one end to form a small triangular center zone. To obtain an accurate assessment of behavioral performance in the Y-maze, objective definitions of arm entries and exits are necessary^13,29^. In this study, we defined the center zone using occupancy maps of the head and tail base positions. Our approach resulted in a substantially larger center zone, thus reducing the false counting of arm entries. Our method is advantageous as it relies purely on the animal’s behavior rather than the physical arrangement of the maze, making it suitable for comparing results across animals of different sizes, including juvenile, and adult animals in the same Y-maze.

The conventional method for calculating the spontaneous alternation rate involves dividing the number of triads (consecutive entries into three different arms) by the number of arm entries minus two^5,9,14^. However, there is disagreement regarding how far animals must go into the arm for it to be considered a visit^13,29^. We systematically varied the arm visit threshold from the arm entry to the arm end to address this issue. We found that the alternation rate remained similar as the threshold value increased up to 0.7 in the GC cohort. This was mainly because the mice explored the arm to the distal end in over 80% of the arm entries. Thus, the choice of an arm visit threshold is not a critical factor in calculating the spontaneous alternation rate in the GC cohort. However, we observed that the alternation rate significantly decreased in the SI cohort as the arm threshold value increased to 0.7. The decrease was significant when the arm visit threshold value exceeded 0.54, indicating the importance of choosing an appropriate value of the arm threshold to reveal impairments in spontaneous alternation behavior.

The reduced spontaneous alternation rate observed in SI mice could potentially be interpreted as a decrease in their tendency to alternate between arms, rather than a decline in short-term memory. However, the novel measure of ‘difference in recency’ we developed in this study provides additional evidence supporting short-term memory impairment. This measure, concerning the difference in recency between the two potential arms to be selected, did not influence the preference of GC mice to choose the correct, less recently visited arm. In contrast, when incorrect arms were chosen, SI mice showed a considerably lower difference in recency values. This observation provides a compelling evidence that the reduced alternation rate can be attributed to an increased volatility in the memory of recently visited arms in SI mice, rather than a general decrease in their preference for a less recently visited arm.

When analyzing the behavior of mice on the Y-maze, reporting the number of arm entries along with the alternation rate has been a standard practice as it reflects locomotive behavior. Previous studies have suggested that when there is no significant difference in the number of arm entries between the control and treatment groups, any decrease in the spontaneous alternation rate is likely due to impaired short-term memory rather than other behavioral changes^8,9,11–13,24–28^. However, the number of arm entries is an indirect measure and may not capture all behavioral changes. In this study, we analyzed various aspects of mice behavior on the Y-maze to assess how different behavioral factors may influence spontaneous alternation behavior. First, we established that intrinsic turning bias of the mice, an element unrelated to memory, had only a marginal influence on the spontaneous alternation rate. Furthermore, our quantitative analyses showed that locomotive behaviors, such as the number and ratio of arm visits and travel distance, were not correlated with the alternation rate in both the GC and SI cohorts. The significant differences we observed between the GC and SI cohorts was the number of arm visits and travel distance, which did not correlate with the spontaneous alternation rate. Therefore, we concluded that the decrease in the alternation rate in the SI cohort was not due to a decrease in locomotion, suggesting a specific impairment in spatial working memory. However, if there is any difference in behavioral factors between the control and treatment groups and the behavioral factors correlate with the alternation rate, one should be cautious in interpreting the results. Our study highlights the importance of quantitative analyses of various behavioral factors to confidently demonstrate an effect on the spontaneous alternation rate.

The beam breaker method can be prone to errors and false detections when animals display bobbing motions at sensor locations. Thus, the previous studies considered successive breaking of multiple beams in the same arm as an arm visit^7,13,15–17^. Similarly, tracking a single body point can lead to errors when the animal bobs or scans its head near the border between neighboring arena zones, resulting in false detection of arm visits. To overcome this limitation, a new arm visit was counted when the position of the animal passed two different locations^29^. In our study, to improve the accuracy of counting arm visits and arm visit sequence, we determined arm entries, and exits, as well as crossing the arm threshold for sufficient arm visits, as the crossing of both the head and tail base across the border. This approach minimizes erroneous detection of border crossings and thus improves accuracy of counting arm visits and the acquisition of arm visit sequences. Considering the rapid advancements in deep learning-based pose estimation techniques^20–23^, tracking multiple body points is highly recommended for more accurate analyses of spontaneous alternation behaviors in Y mazes.

Although the negative impact of social isolation on memory has garnered significant attention, previous studies have focused on examining the effects of long-term social isolation^39–41^. However, the recent surge of involuntary social isolation caused by the Covid-19 pandemic has sparked increasing interest in the adverse effects of short-term social isolation^30,31,42,43^. The consequences of acute social isolation on social recognition memory in rodents have been well documented. While group-housed mice exhibit long-term social recognition memory that lasts over a day, social isolation for 24 h significantly impairs their long-term social memory^34–38^. However, their short-term social memory up to 1 h remains intact following acute social isolation^34–38^. In addition, acute social isolation promotes social interaction^42,44–46^, whereas it exerts no effect on objection recognition memory^37^. In this study, our quantitative approach was employed to evaluate the impact of 24-h social isolation on spatial working memory, another form of short-term memory. The results revealed that the SI cohort displayed a reduced spontaneous alternation rate, indicating impaired short-term spatial memory. Thus, this study has expanded our understanding of the effects of acute social isolation on the spatial domain.

## Methods

### Animals

Behavioral data were collected from 52 10-week-old C57BL/6 J male mice (The Jackson Laboratory). The mice were maintained at 20–22 °C with humidity between 40 and 60% on a 12-h light/dark cycle with food and water ad libitum. All procedures used in this work were approved by the Institutional Animal Care and Use Committee of the Institute for Basic Science (Daejeon, South Korea). All experiments were performed in accordance with the institutional guidelines. All authors complied with the ARRIVE guidelines.

### Social isolation

For group-housed control subjects, 24 mice were cohoused with conspecifics of four mice per cage. To simulate acute social isolation, 28 mice were subjected to 24 h of single-housing conditions. Other factors, except housing conditions, were controlled to be identical for the GC cohort and the SI cohort.

### Y-maze spontaneous alternation test

The Y-maze apparatus was made out of acrylic plastic with a color of black or gray. The floor was 4 cm in width, each arm was 36 cm long, and the walls were 15.5 cm in height. Behavioral data were recorded using a Samsung CCD camera (Samsung) or a Point-Grey camera (FLIR). The apparatus was enclosed in a soundproof box, which measured 80 × 70 × 145 cm^3^ and was illuminated with 10 lux. The behavioral experiments were conducted between 14:00 and 18:00, during the light phase of the light/dark cycle. Due to the implementation of social isolation, where physical contact was avoided during the isolation period, we deliberately refrained from performing any handling procedures. Prior to initiating the behavioral tests, we allowed the animals to acclimate in the testing room for a minimum of 30 min. A white noise generator (Med Associates Inc., VT, USA) was utilized to mask the background sound in the testing room, generating 65 dB of white noise. The mice were placed in the Y-maze for approximately 8 min, and the data used for analysis were 7 min and 45 s in length for all subjects. Once a test was completed, we carefully removed the mouse from the maze and proceeded to clean the maze using 70% ethanol followed by distilled water. We allowed a 5-min interval before testing the next mouse to ensure the elimination of any lingering odors. All other experimental conditions remained consistent between the group-housed control and socially isolated mice.

### Animal tracking

The body parts of the mice were tracked using the open-source software DeepLabCut^12^. The tracked body parts were the nose, left, and right ears, neck, and tail base. The head position was calculated as the mean coordinates of the nose, left, and right ears, and neck. Frames extracted automatically using the k-means algorithm were labeled manually, which were then used to train the DeepLabCut network.

### Occupancy map

The distance between the head or tail base and the center of the maze was normalized by arm length. The histogram of the distance values was pooled across different mice.

### Determination of arm visit

An entry and an exit of an arm were defined as when both the head and the tail base crossed the boundary of the center zone defined by the occupancy map of the head and tail base. For example, once both the head, and tail base cross the boundary into the arm, the arm visit continues until both the head and tail base cross the boundary back into the center zone. The duration of an arm visit was defined as the time between an arm entry and the following arm exit. If the head and tail base positions crossed the arm threshold during an arm visit, it was considered a sufficient arm visit.

### Calculation of the spontaneous alternation rate

Each arm choice was classified correct, incorrect, or neutral. If the last and second last-visited arms were different and the current choice was different from those two arms, then it was considered a correct choice. If the mice chose the other arms, the choice was considered incorrect. If the last and second last-visited arms were the same, i.e., the mice consecutively visited the same arm twice, then the current arm choice was considered neutral. The spontaneous alternation rate was calculated as the number of correct choices divided by the total number of correct and incorrect choices.

### Calculation of the chance-level spontaneous alternation rate

Arm choice bias was calculated as the absolute difference between the ratio between clockwise and counterclockwise choices and 0.5, the ratio that would be acquired if the subjects make the same number of clockwise and counterclockwise choices. Based on the mean number of arm visits and the calculated arm choice bias, 1000 iterations of alternation simulations were performed. The correct rate derived from the simulations was used to calculate the chance-level spontaneous alternation rate.

### Statistics

All data analyses were performed with MATLAB (MathWorks) with custom codes. A *p* value < 0.05 was used as the criterion for statistical significance. All data were expressed as mean ± SEM.

## Data Availability

Sample trajectory data and the analysis code are available at https://github.com/doyunleelab/Y-maze. Additional data are available upon request to the corresponding author.
